# Effect Analysis of GNSS/INS Processing Strategy for Sufficient Utilization of Urban Environment Observations

**DOI:** 10.3390/s21020620

**Published:** 2021-01-17

**Authors:** Bo Shi, Mengke Wang, Yunpeng Wang, Yuntian Bai, Kang Lin, Fanlin Yang

**Affiliations:** 1 College of Geodesy and Geomatics, Shandong University of Science and Technology, Qingdao 266000, China; shibo@sdust.edu.cn (B.S.); mengkewang@sdust.edu.cn (M.W.); skdwangyp@163.com (Y.W.); baitian123@sdust.edu.cn (Y.B.); linkang95@sdust.edu.cn (K.L.); 2 Key Laboratory of Ocean Geomatics, Ministry of Natural Resources of China, Qingdao 266000, China

**Keywords:** urban canyon environment, differential global navigation satellite system/inertial navigation system, tightly coupled, aided inertial navigation system, integer ambiguity resolution, Rauch–Tung–Striebel smoothing

## Abstract

The occlusion of buildings in urban environments leads to the intermittent reception of satellite signals, which limits the utilization of observations. This subsequently results in a decline of the positioning and attitude accuracy of Global Navigation Satellite System (GNSS)/Inertial Navigation System (INS) integrated system (GNSS/INS). This study implements a smooth post-processing strategy based on a tightly coupled differential GNSS/INS. Specifically, this strategy used the INS-estimated position to reinitialize integer ambiguity. The GNSS raw observations were input into the Kalman filter to update the measurement. The Rauch–Tung–Striebel smoothing (RTSS) algorithm was used to process the observations of the entire period. This study analyzed the performance of loosely coupled and tightly coupled systems in an urban environment and the improvement of the RTSS algorithm on the navigation solution from the perspective of fully mining the observations. The experimental results of the simulation data and real data show that, compared with the traditional tightly coupled processing strategy which does not use INS-aided integer ambiguity resolution and RTSS algorithm, the strategy in this study sufficiently utilized INS observations and GNSS observations to effectively improve the accuracy of positioning and attitude and ensure the continuity of navigation results in an obstructed environment.

## 1. Introduction

With the development of the geospatial information service industry, the demand for rapid and accurate geospatial information access has increased. As an important means of obtaining spatial position information, Global Navigation Satellite System (GNSS)/Inertial Navigation System (INS) technology has wide application prospects in mobile measurement [1], autonomous driving [2], and intelligent service [3] fields. GNSS has high long-term absolute positioning accuracy, but its anti-interference ability is weak, and its sampling rate is low. INS can navigate autonomously without being affected by the external environment, and it exhibits high short-term accuracy and sampling rate, but it has one significant problem by which errors accumulate over time [4,5]. Therefore, GNSS/INS integration can achieve the complementary advantages of high-precision positioning and attitude determination. GNSS/INS integration is typically applied in urban environments, which are extremely complex, with dense high-rise buildings, viaducts, tunnels, and other infrastructure. Of these, the most typical environment is the urban canyon, which is formed by dense building blocks and tall trees. In this environment, the GNSS signal suffers frequent lock loss, and the existing observation data are not fully utilized and do not meet the demand for high-precision positioning and pose determination. Therefore, it is necessary to analyze the performance of GNSS/INS to improve the accuracy of positioning and attitude determination in this environment.

In recent years, domestic and foreign researchers have investigated partial and complete GNSS signal lock of loss. The improved methods mostly increased the accuracy of the integrated system by adding observations; these can be divided according to whether additional sensors are added or not. One method involves maintaining heading and speed updates using observations from external sensors or equipment, such as odometers and laser scanners. In [6], GNSS, INS, and an odometer are integrated into a single system, and speed information measured by the odometer is used to assist the INS with navigation and calculation when the GNSS signal is interrupted. In [7], GNSS, INS, and light detection and ranging (LiDAR) technology observations are fused using an extended Kalman filter. When there are no GNSS observations, LiDAR observations are used to assist the INS in estimating and compensating for the sensor error. In [8], based on graph optimization theory, the GNSS position, inertial measurement unit (IMU) pre-integration result, and relative pose matched by the LiDAR scan are fused, which limits the horizontal position error during satellite signal interruption. Although adding sensors can partially improve the accuracy of the GNSS/INS combined system in a satellite denied environment, it also increases the cost and complexity of the system. Another method is to construct non-holonomic constraints based on the state and law of carrier motion to suppress the drift error of the INS inertial device. In [9], a velocity constraint equation was constructed under the assumption that the carrier is always in contact with the ground and does not slip; an elevation constraint was proposed based on small changes in the carrier elevation over a short period of time. By increasing the redundancy of the measured values, the accumulation of inertial navigation errors is controlled, and the speed constraint can provide higher precision pose results than those of the height constraint. In [10], a heading angular velocity constraint that can enhance dynamic vehicle navigation accuracy was proposed. Analyzing non-holonomic constraint observations provides a strategy for selecting observations for different constraints. This method avoids the addition of more sensors by constructing a constrained observation vector with fixed observations. The actual observations are not constant due to changes in terrain and carrier motion, and as a result, improved navigation accuracy cannot be guaranteed. In addition, this method can only be used in a carrier movement environment that meets the limited constraints.

Scholars have also studied how to improve the positioning and attitude accuracies of integrated systems in an urban environment using GNSS/INS integration methods and filtering models without any additional observations. Currently, GNSS/INS integration is primarily divided into three methods: loosely coupled, tightly coupled, and deeply coupled. The deeply coupled method combines GNSS and INS observations and GNSS signal tracking into one filter and uses GNSS to track satellite signals through INS. It is an integration on the hardware level, which is difficult to implement and has not been widely used [11,12]. The loosely coupled method integrates GNSS with assisted INS, which has the advantage of a simple structure. However, when the number of observation satellites is less than four, the Kalman filter stops working, and the integrated system degenerates into a single system with only INS [13]. The tightly coupled method is a form of mutual assistance between the GNSS and INS. It inputs the original GNSS observations as measured values into a Kalman filter to correct INS navigation errors and inertial device errors. INS simultaneously aid GNSS for cycle slip detection and repair [14] and integer ambiguity resolution (IAR) [15]. In [16], recognizing the high cost of high-precision inertial navigation, a low-cost sensor micro-electro-mechanical system IMU is used for tight coupling. The improvement of tightly coupled positioning performance is analyzed through experiments in three typical actual urban environments. Compared with the loosely coupled systems, tightly coupled systems exhibit superior robustness and are more suitable for satellite signal multi-occlusion environments, such as urban canyons. In [17], by simulating an observation environment with an insufficient number of satellites, the effect of different satellite numbers on the performance of the Precise Point Positioning (PPP)/INS tightly coupled with non-holonomic constraints is analyzed, and it was concluded that the greater the number of observation satellites, the better the positioning accuracy.

Differential GNSS (DGNSS) based on the carrier phase is a commonly used high-precision positioning method. Under the premise that the integer ambiguity is fixed correctly, the DGNSS/INS can achieve centimeter-level positioning accuracy. In the DGNSS/INS tightly coupled model, the integer ambiguity solution is primarily divided into two methods: a centralized Kalman filter [18] and a decentralized Kalman filter [19]. In [20], a tightly coupled real-time kinematic (RTK)/INS algorithm based on a centralized Kalman filter was designed, and a float solution update was used to avoid difficulties in fixing the ambiguity when the number of satellites was less than four. This algorithm can effectively use less than four carrier phase observations to limit the accumulation of inertial device errors and quickly resolve integer ambiguity. However, a centralized filter expands integer ambiguity into the state vector and estimates it with INS errors. Although this method can constrain the ambiguity of adjacent epochs, it increases the filter order and calculation amount. Additionally, for this filter type, the ambiguity at the previous moment affects the current state estimation, and the accuracy and stability of the filter can be seriously affected when the GNSS signal is frequently blocked and the fault tolerance is poor [21]. Decentralized Kalman filtering involves placing the ambiguity parameters into a sub-filter to be individually fixed and then uses accurate carrier phase observations to measure and update the primary filter. In [22], the ambiguity solution and the estimation of the navigation state and inertial device errors are realized using a dual Kalman filter. However, it is difficult to fix the ambiguity in this model when there are fewer than four satellites, which directly affects the accuracy of the navigation solution. In addition, two filters are more difficult to implement. [23] tackles the issues of large computational burden and poor robustness in tightly coupled multi-constellation, multi-frequency, and multi-type observation models by utilizing a sequential Kalman filter for data fusion between the two sensors. For GNSS channel failure detection and prevention, a robust estimation method based on the Gaussian test was proposed. In addition, optimal smoothing based on the Kalman filter can use measurement data to provide the optimal estimate for a certain measurement period. Therefore, when real-time processing is not required, a post-processing smoothing algorithm can make full use of the period observations to improve the pose accuracy [24]. Currently, commonly used post-processing smoothing algorithms include the Rauch–Tung–Striebel smoothing (RTSS) algorithm and the forward-backward smoothing (FBS) algorithm. FBS requires backward filtering, which increases the amount of data storage required, and it only improves the position accuracy. In contrast, in the tightly coupled DGNSS/INS, the double-difference ambiguity is easier to fix, and the accuracy of the position result meets the requirements of surveying and mapping applications. Therefore, there is no need to further process the position solution through FBS [25]. RTSS only requires two steps, forward filtering and backward smoothing, and its data storage capacity is small and easy to program. However, when the ambiguity parameter is estimated as a part of the state vector, it is difficult to improve the accuracy of the navigation solution during initialization and reconvergence [26].

Currently, research on the tightly coupled GNSS/INS in an urban environment is focused on improving the filtering model and adding redundant observations. There are few studies regarding making full use of available observations in an environment with frequent losses of satellite signals. Therefore, this study aims to implement a smooth post-processing strategy based on a tightly coupled carrier phase DGNSS/INS. This strategy utilizes a decentralized filter to fix integer ambiguity, thereby avoiding the existing problem of the RTSS algorithm in the tightly coupled GNSS/INS. After the GNSS signal is interrupted, the ambiguity is quickly fixed by the INS in the ambiguity initialization stage. Then, the result is further smoothed by the RTSS. This study first analyzes the positioning and attitude determination performance of the loosely coupled and tightly coupled DGNSS/INS when the number of observable satellites is insufficient through a simulated satellite lock-loss experiment, and verifies the effectiveness of the RTSS in improving the pose accuracy in an occlusion environment. Then, field experiments are used to verify method effectiveness. The results show that the proposed strategy can effectively improve the pose accuracy even during the intermittent reception of satellite signals. It can provide a more reliable and high-precision solution for navigation and positioning tasks in challenging environments.

## 2. Materials and Methods

### 2.1. Differential Tightly Coupled GNSS/INS

**Figure 1:**
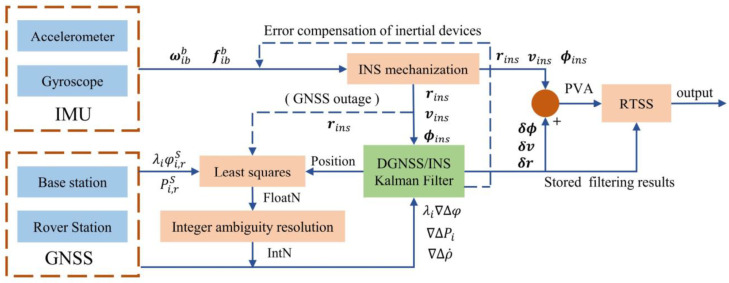
Tightly coupled data processing strategy. (Note.$\;f_{ib}^{b}$ is specific force measured by the accelerometer.$\;\omega_{ib}^{b}$ is the angular rate measured by the gyroscope. $\lambda_{i}\varphi_{i,r}^{S}$ and $P_{i,r}^{S}$ are raw carrier phase and pseudorange observations, respectively. $r_{ins}$, $v_{ins}$ and $\phi_{ins}$ are position, velocity and attitude estimated by INS, respectively. δϕ, δv and δr are the correction of position, velocity and attitude, respectively. $\lambda_{i}\nabla \Delta \varphi$, $\nabla \Delta P_{i}$ and $\nabla \Delta \dot{\rho }$ are double difference observations. FloatN represent ambiguity float solution and IntN represent inter ambiguity solution. PVA means position, velocity, and attitude.

In the tightly coupled DGNSS/INS extended Kalman filter model, Correct IAR is the premise driving high-precision carrier phase positioning. Using decentralized filters, the tightly coupled model is divided into two parts: a tightly coupled filter module and an IAR module, which lowers the dimensionality of the filter matrix and improves fault tolerance. The primary function of the IAR module is to estimate the integer ambiguity parameters. When a new satellite is observed, the position information output by the filter assists with IAR. After the satellite signal interruption ends and the integer ambiguity must be fixed again, the position information calculated by the INS is used to assist the reinitialization of the integer ambiguity. The tightly coupled filter module exhibits the simplest system model because the ambiguity is not extended to the state vector. The state vector, covariance matrix, INS pose, and other sequentially stored information are inputted into the RTSS module to optimally smooth the entire filtering process. Figure 1 displays the tightly coupled data processing strategy used in this study.

#### 2.1.1. System Model.

The system model is based on the INS error an Earth-centered Earth-fixed (ECEF) frame, the error equation in the ECEF is selected to construct the system model more conveniently. The INS psi-angle error model in the ECEF is given as [27]

(1) $$\begin{array}{c} \left(\begin{array}{c} \dot{\phi^{e}}\\ \begin{array}{c} \delta {\dot{v}}^{e}\\ \delta {\dot{r}}^{e} \end{array} \end{array}\right)=\left(\begin{array}{c} -\left[\omega_{ie}^{e}\times \right]\phi^{e}-C_{b}^{e}\delta \omega_{ib}^{b}\\ \begin{array}{c} C_{b}^{e}\delta f_{ib}^{b}+\left[\left(C_{b}^{e}f_{ib}^{b}\right)\times \right]\phi^{e}-2\left[\omega_{ie}^{e}\times \right]\delta v^{e}+\delta g^{e}\\ \delta v^{e} \end{array} \end{array}\right) \end{array}$$

where · is the differential symbol; i, e, and b represent the Earth-centered inertial frame, ECEF frame, and IMU body frame, respectively; (a)× represents a skew-symmetrical matrix composed of vector a; ϕ, $\delta v^{e}$ and $\delta r^{e}$ are the attitude error vector, velocity error vector, and position error vector, respectively; $C_{b}^{e}$ is the direction cosine matrix of the IMU body frame relative to the ECEF; $\omega_{ie}^{e}$ is the earth rotation;$\;\delta g^{e}$ is the gravity error vector in e-frame; $f_{ib}^{b}$ is specific force vector; and $\delta f_{ib}^{b}$ and $\delta \omega_{ib}^{b}$ are the measurement error for accelerometers and gyroscope, respectively. The specific derivation process of Equation (1) can be found in Appendix A.

The tactical-grade IMU used in this study does not consider the scale factor error or cross-coupling error, but the random errors of inertial devices need to be considered. The IMU measurement error model is as follows

(2) $$\begin{array}{c} \begin{array}{c} \delta \omega_{ib}^{b}=\delta b_{g}+w_{\omega }\\ \delta f_{ib}^{b}=\delta b_{a}+w_{f} \end{array} \end{array}$$

where $w_{\omega }$ and $w_{f}$ are white noise of gyroscope and accelerometer, respectively; $\delta b_{g}$ and $\delta b_{a}$ are the gyroscope and accelerometer bias errors respectively, which can be modeled as first-order Gauss–Markov processes:

(3) $$\begin{array}{c} \begin{array}{c} \delta {\dot{b}}_{g}=-\frac{1}{T_{g}}\delta b_{g}+w_{b}\\ \delta {\dot{b}}_{a}=-\frac{1}{T_{a}}\delta b_{a}+w_{a} \end{array} \end{array}$$

where $T_{g}$ and $T_{a}$ are the correlation time of gyroscope and accelerometer, respectively; $w_{b}$ and $w_{a}$ are the driven white noise of gyroscope and accelerometer, respectively.

Based on Equations (2) and (3), the bias error is expanded to the state parameters for estimation. The system equation can be expressed as follows:

(4) $$\begin{array}{c} \dot{x}\left(t\right)=F\left(t\right)x\left(t\right)+G\left(t\right)w\left(t\right) \end{array}$$

where,

(5) $$\begin{array}{c} x={\left[{\left(\phi \right)}^{T}\;{\left(\delta r^{e}\right)}^{T}\;{\left(\delta v^{e}\right)}^{T}\;{\left(\delta b_{g}\right)}^{T}\;{\left(\delta b_{a}\right)}^{T}\right]}^{T} \end{array}$$

F is the continuous system state transition matrix; G is the continuous system noise distribution matrix; w is the system noise vector. The specific form of the above matrix can be found in [28].

Equation (4) is a continuous dynamics equation, and it must be discretized as follows:

(6) $$\begin{array}{c} x_{k}=\Phi_{k/k-1}x_{k-1}+w_{k-1} \end{array}$$

The specific form of each matrix in Equation (6) can be found in [29]. In this study, the power spectral density matrix of the system noise matrix is set up by angle random walk and speed random walk in the inertial navigation manual [30].

#### 2.1.2. Measurement Model

GNSS uses satellites in orbit to transmit radio signals to provide users with position information through passive ranging. The basic observation equation is as follows [31]:

(7) $$\begin{array}{cc} \lambda_{i}\varphi_{i,r}^{S}\left(t_{r}\right) & =\rho_{r}^{S}\left(t_{r},t^{S}\right)-c\left(dT_{r}-dT^{S}\right)-\lambda_{i}N_{i,r}^{S}-I_{i,r}^{S}+T_{r}^{S}-\left(d_{i,r,\varphi }-D_{i,\varphi }^{S}\right)\\ & +M_{ir,\lambda \varphi }^{S}+R_{r}^{S}+\varepsilon_{i,r,\lambda \varphi }^{S}\\ P_{i,r}^{S}\left(t_{r}\right) & =\rho_{r}^{S}\left(t_{r},t^{s}\right)-c\left(dT_{r}-dT^{S}\right)+I_{i,r}^{S}+T_{r}^{S}-\left(d_{i,r,P}-D_{i,P}^{S}\right)\\ & +M_{i,r,P}^{S}+R_{r}^{S}+\varepsilon_{i,r,P}^{S}\\ {\dot{P}}_{i,r}^{S} & =-\frac{c}{f_{i}}D_{i} \end{array}$$

where i is the signal frequency; $\lambda_{i}$ is the wavelength of the carrier; $f_{i}$ is the frequency of the carrier; r is the receiver number; s is the satellite number; $P_{i,r}^{S}\left(t_{r}\right)$ is the code measurement of the pseudorange observations; $\lambda_{i}\varphi_{i,r}^{S}\left(t_{r}\right)$ is the carrier phase observations; ${\dot{P}}_{i,r}^{S}$ is the pseudorange rate; $N_{i,r}^{S}$ is the integer ambiguity; $\rho_{r}^{S}\left(t_{r},t^{S}\right)$ is the geometric distance from the satellite to the receiver; c is the speed of light in vacuum; $dT_{r}$ is the receiver clock error;$\;dT^{S}$ is the satellite clock error; $I_{i,r}^{S}$ is the ionospheric delay; $T_{r}^{S}$ is the tropospheric delay; $d_{i,r,\varphi }$ and $d_{i,r,P}$ are the receiver hardware phase and code delay, respectively;$\;D_{i,\varphi }^{S}$ and $D_{i,P}^{S}$ are the satellite hardware phase and code delay, respectively; $M_{i,r,P}^{S}$ is the multipath effect of the code measurement pseudorange; $R_{r}^{S}$ is the multipath effect; $\varepsilon_{i,r}^{S}$ is the observation error; $D_{i}$ is the Doppler frequency shift, where $D_{i}=\lambda_{i}\frac{\left[P^{S}-P_{r}\right]\cdot \left[V_{r}-V^{S}\right]}{\rho }$; ρ is the geometric distance between the satellite and receiver; $P^{S}$ and $P_{r}$ are the satellite and station coordinates, respectively; and $V_{r}$ and $V^{S}$ are the satellite and station velocities, respectively.

There are a variety of error terms in Equation (7), which inhibit integer characteristic maintenance by non-difference ambiguity; thus, the accuracy of the solution directly using the non-difference observation equation is limited. The use of double-difference observations for positioning calculations can eliminate error items such as the receiver clock error, satellite clock error, and receiver and satellite hardware phase delay, and it can greatly reduce the impact of ionospheric and tropospheric delays on the observations to ensure the integer characteristics of double-difference ambiguity [31]. The observation equation of the double difference between the stations is as follows:

(8) $$\begin{array}{cc} \lambda_{i}\nabla \Delta \varphi_{i,r_{1,2}}^{S_{1,2}} & =\nabla \Delta \rho_{r_{1,2}}^{S_{1,2}}-\lambda_{i}\nabla \Delta N_{i,r_{1,2}}^{S_{1,2}}-\nabla \Delta I_{i,r_{1,2}}^{S_{1,2}}+\nabla \Delta T_{r_{1,2}}^{S_{1,2}}+\nabla \Delta M_{i,r_{1,2},\lambda \varphi }^{S_{1,2}}\\ & +\nabla \Delta d_{r_{1,2},other}^{S_{1,2}}+\nabla \Delta \varepsilon_{i,r_{1,2},\lambda \varphi }^{S_{1,2}}\\ \nabla \Delta P_{i,r_{1,2}}^{S_{1,2}} & =\nabla \Delta \rho_{r_{1,2}}^{S_{1,2}}-\nabla \Delta I_{i,r_{1,2}}^{S_{1,2}}+\nabla \Delta T_{r_{1,2}}^{S_{1,2}}+\nabla \Delta M_{i,r_{1,2,},P}^{S_{1,2}}\\ & +\nabla \Delta d_{r_{1,2},others}^{S_{1,2}}+\nabla \Delta \varepsilon_{i,r_{1,2},P}^{S_{1,2}}\\ \nabla \Delta {\dot{\rho }}_{i,r_{1,2}}^{S_{1,2}} & =-\lambda_{i}\nabla \Delta D_{i,r_{1,2}}^{S_{1,2}}+\nabla \Delta \varepsilon_{\dot{\rho }} \end{array}$$

where ∇Δ is the double-difference operator; $r_{1,2}$ represents the base station and rover, respectively; and $S_{1,2}$ represents the two satellites simultaneously observed by $r_{1,2}$.

Simplified as:

(9) $$\begin{array}{cc} \lambda_{i}\nabla \Delta \varphi & =\nabla \Delta \rho -\lambda_{i}\nabla \Delta N_{i}-\nabla \Delta I_{i}+\nabla \Delta T+\nabla \Delta M_{i,\lambda \varphi }+\nabla \Delta d_{others}+\nabla \Delta \varepsilon_{i,\lambda \varphi }\\ \nabla \Delta P_{i} & =\nabla \Delta \rho -\nabla \Delta I_{i}+\nabla \Delta T+\nabla \Delta M_{i,p}+\nabla \Delta d_{others}+\nabla \Delta \varepsilon_{i,\lambda \varphi }\\ \nabla \Delta \dot{\rho } & =-\lambda_{i}\nabla \Delta D_{i}+\nabla \Delta \varepsilon_{\dot{\rho }} \end{array}$$

when the differential GNSS and INS are integrated, the approximate values of ∇Δρ and $\nabla \Delta D_{i}$ are derived from the position and velocity outputs by the INS. Equation (9) is the basis for constructing the tightly coupled extended Kalman filter observation model in this study.

The position and speed corrected by the lever arm value are considered in Equation (9) and linearized to obtain the double-difference observation equation:

(10) $$\left[\begin{array}{c} \lambda_{1}\nabla \Delta \varphi_{1}-\nabla \Delta \rho_{INS}\\ \nabla \Delta P_{1}-\nabla \Delta \rho_{INS}\\ \nabla \Delta \dot{\rho_{1}}-\nabla \Delta v_{\rho ,INS} \end{array}\right]=\left[\begin{array}{ccccc} H_{1} & H_{2} & 0 & 0 & 0\\ H_{1} & H_{2} & 0 & 0 & 0\\ H_{3} & 0 & H_{4} & H_{5} & 0 \end{array}\right]\left[\begin{array}{c} \phi \\ \delta r^{e}\\ \delta v^{e}\\ b_{g}\\ b_{a} \end{array}\right]+\left[\begin{array}{c} \nabla \Delta \varepsilon_{\Phi_{1}}\\ \nabla \Delta \varepsilon_{P_{1}}\\ \nabla \Delta \varepsilon_{{\dot{\rho }}_{1}} \end{array}\right]$$

where $\nabla \Delta \varphi_{1}$, $\nabla \Delta P_{1}$, and $\nabla \Delta \dot{\rho_{1}}$ are the carrier phase, pseudorange, and pseudorange rate double-difference observations, respectively; $\nabla \Delta \rho_{INS}$ and $\nabla \Delta v_{\rho ,INS}$ are the distance and speed double-difference observation values calculated by INS, respectively; ∇Δε is the observation error vector; and H is the coefficient matrix of the state parameter given as

$$H_{1}=\left[\begin{array}{ccc} \begin{array}{c} \begin{array}{c} \nabla \Delta p_{\Phi ,1}\\ \nabla \Delta p_{\Phi ,2} \end{array}\\ \vdots \\ \nabla \Delta p_{\Phi ,n} \end{array} & \begin{array}{c} \begin{array}{c} \nabla \Delta h_{\Phi ,1}\\ \nabla \Delta h_{\Phi ,2} \end{array}\\ \vdots \\ \nabla \Delta h_{\Phi ,n} \end{array} & \begin{array}{c} \begin{array}{c} \nabla \Delta i_{\Phi ,1}\\ \nabla \Delta i_{\Phi ,2} \end{array}\\ \vdots \\ \nabla \Delta i_{\Phi ,n} \end{array} \end{array}\right]\;H_{2}=\left[\begin{array}{ccc} \begin{array}{c} \begin{array}{c} \nabla \Delta l_{\Phi ,1}\\ \nabla \Delta l_{\Phi ,2} \end{array}\\ \vdots \\ \nabla \Delta l_{\Phi ,n} \end{array} & \begin{array}{c} \begin{array}{c} \nabla \Delta m_{\Phi ,1}\\ \nabla \Delta m_{\Phi ,2} \end{array}\\ \vdots \\ \nabla \Delta m_{\Phi ,n} \end{array} & \begin{array}{c} \begin{array}{c} \nabla \Delta n_{\Phi ,1}\\ \nabla \Delta n_{\Phi ,2} \end{array}\\ \vdots \\ \nabla \Delta n_{\Phi ,n} \end{array} \end{array}\right]\;H_{3}=\left[\begin{array}{ccc} \begin{array}{c} \begin{array}{c} \nabla \Delta p_{\dot{\rho },1}\\ \nabla \Delta p_{\dot{\rho },2} \end{array}\\ \vdots \\ \nabla \Delta p_{\dot{\rho },n} \end{array} & \begin{array}{c} \begin{array}{c} \nabla \Delta h_{\dot{\rho },1}\\ \nabla \Delta h_{\dot{\rho },2} \end{array}\\ \vdots \\ \nabla \Delta h_{\dot{\rho },n} \end{array} & \begin{array}{c} \begin{array}{c} \nabla \Delta i_{\dot{\rho },1}\\ \nabla \Delta i_{\dot{\rho },2} \end{array}\\ \vdots \\ \nabla \Delta i_{\dot{\rho },n} \end{array} \end{array}\right]$$

$$H_{4}=\left[\begin{array}{ccc} \begin{array}{c} \begin{array}{c} \nabla \Delta l_{\dot{\rho },1}\\ \nabla \Delta l_{\dot{\rho },2} \end{array}\\ \vdots \\ \nabla \Delta l_{\dot{\rho },n} \end{array} & \begin{array}{c} \begin{array}{c} \nabla \Delta m_{\dot{\rho },1}\\ \nabla \Delta m_{\dot{\rho },2} \end{array}\\ \vdots \\ \nabla \Delta m_{\dot{\rho },n} \end{array} & \begin{array}{c} \begin{array}{c} \nabla \Delta n_{\dot{\rho },1}\\ \nabla \Delta n_{\dot{\rho },2} \end{array}\\ \vdots \\ \nabla \Delta n_{\dot{\rho },n} \end{array} \end{array}\right]\;H_{5}=\left[\begin{array}{ccc} \begin{array}{c} \begin{array}{c} \nabla \Delta u_{\dot{\rho },1}\\ \nabla \Delta u_{\dot{\rho },2} \end{array}\\ \vdots \\ \nabla \Delta u_{\dot{\rho },n} \end{array} & \begin{array}{c} \begin{array}{c} \nabla \Delta t_{\dot{\rho },1}\\ \nabla \Delta t_{\dot{\rho },2} \end{array}\\ \vdots \\ \nabla \Delta t_{\dot{\rho },n} \end{array} & \begin{array}{c} \begin{array}{c} \nabla \Delta g_{\dot{\rho },1}\\ \nabla \Delta g_{\dot{\rho },2} \end{array}\\ \vdots \\ \nabla \Delta g_{\dot{\rho },n} \end{array} \end{array}\right]$$

The details of above matrix are given as follows:

(11) $$p_{\Phi }\;=\frac{\Delta y{\left({\tilde{C}}_{b}^{e}l^{b}\right)}_{z}-\Delta z{\left({\tilde{C}}_{b}^{e}l^{b}\right)}_{y}}{\rho_{INS}}\;h_{\Phi }=\frac{\Delta z{\left({\tilde{C}}_{b}^{e}l^{b}\right)}_{x}-\Delta x{\left({\tilde{C}}_{b}^{e}l^{b}\right)}_{z}}{\rho_{INS}}\;i_{\Phi }=\frac{\Delta x{\left({\tilde{C}}_{b}^{e}l^{b}\right)}_{y}-\Delta y{\left({\tilde{C}}_{b}^{e}l^{b}\right)}_{x}}{\rho_{INS}}\;l_{\Phi }=\frac{\Delta x}{\rho_{INS}}\;m_{\Phi }=\frac{\Delta y}{\rho_{INS}}\;n_{\Phi }=\frac{\Delta z}{\rho_{INS}}\;p_{\dot{\rho }}=-\frac{\Delta xA_{11}+\Delta yA_{21}+\Delta zA_{31}}{\rho_{INS}}\;h_{\dot{\rho }}=-\frac{\Delta xA_{12}+\Delta yA_{22}+\Delta zA_{32}}{\rho_{INS}}\;i_{\dot{\rho }}=-\frac{\Delta xA_{13}+\Delta yA_{23}+\Delta zA_{33}}{\rho_{INS}}\;u_{\dot{\rho }}=-\frac{\Delta xB_{11}+\Delta yB_{21}+\Delta zB_{31}}{\rho_{INS}}\;t_{\dot{\rho }}=-\frac{\Delta xB_{12}+\Delta yB_{22}+\Delta zB_{32}}{\rho_{INS}}\;g_{\dot{\rho }}=-\frac{\Delta xB_{13}+\Delta yB_{23}+\Delta zB_{33}}{\rho_{INS}}\;l_{\dot{\rho }}=\frac{-\Delta x}{\rho_{INS}}\;m_{\dot{\rho }}=\frac{-\Delta y}{\rho_{INS}}\;n_{\dot{\rho }}=\frac{-\Delta z}{\rho_{INS}}$$

In Equation (11), $\rho_{INS}$ is the distance between the satellite and receiver; Δx, Δy, Δz is the coordinate difference between satellite and receiver; $\left(x^{s},y^{s},z^{s}\right)$ are the satellite coordinates; $\left(\tilde{x},\tilde{y},\tilde{z}\right)$ are the receiver coordinates estimated by INS; $l^{b}$ is lever arm vector; where

$$\begin{array}{c} \begin{array}{c} \begin{array}{c} \Delta x=x^{s}-\tilde{x}\\ \Delta y=y^{s}-\tilde{y}\\ \Delta z=z^{s}-\tilde{z} \end{array}\\ \rho_{INS}=\sqrt{{\left(\Delta x\right)}^{2}+{\left(\Delta y\right)}^{2}+{\left(\Delta z\right)}^{2}} \end{array}\\ A=\left[\omega_{ie}^{e}\times \right]\left[\left({\tilde{C}}_{b}^{e}l^{b}\right)\times \right]+\left[\left({\tilde{C}}_{b}^{e}\left(l^{b}\times {\hat{\omega }}_{ib}^{b}\right)\right)\times \right]\\ B={\tilde{C}}_{b}^{e}\left[l^{b}\times \right] \end{array}$$

Further, the observations from Equation (10) can be expressed as

(12) $$\begin{array}{c} z_{k}=H_{k}x_{k}+v_{k} \end{array}$$

For the covariance matrix of the observation noise $v_{k}:$

$$\begin{array}{c} R_{k}=diag\left(\begin{array}{ccc} DR_{\Phi_{1}}D^{T} & DR_{P_{1}}D^{T} & DR_{{\dot{\rho }}_{1}}D^{T} \end{array}\right) \end{array}$$

where D is the single difference matrix,

$$D=[\begin{array}{lllll} -1 & 1 & 0 & \cdots & 0\\ -1 & 0 & 1 & \cdots & 0\\ \vdots & \vdots & \vdots & \ddots & \vdots \\ -1 & 0 & 0 & \cdots & 1 \end{array}]$$

$R_{\Phi_{1}}$,$\;R_{P_{1}}$,$\;R_{{\dot{\rho }}_{1}}$ represent the measurement noise covariance of the carrier phase, pseudorange and pseudorange rate, respectively.

$$R_{\Phi_{1}}=diag\left(\begin{array}{cccc} 2\sigma_{\Phi_{1,1}}^{2} & 2\sigma_{\Phi_{1,2}}^{2} & \cdots & 2\sigma_{\Phi_{1,n}}^{2} \end{array}\right);\;R_{P_{1}}=diag\left(\begin{array}{cccc} 2\sigma_{P_{1,1}}^{2} & 2\sigma_{P_{1,2}}^{2} & \cdots & 2\sigma_{P_{1,n}}^{2} \end{array}\right);\;R_{{\dot{\rho }}_{1}}=diag\left(\begin{array}{cccc} 2\sigma_{{\dot{\rho }}_{1,1}}^{2} & 2\sigma_{{\dot{\rho }}_{1,2}}^{2} & \cdots & 2\sigma_{{\dot{\rho }}_{1,n}}^{2} \end{array}\right);$$

where

$$\sigma_{\Phi_{1}}=\frac{\sigma_{\Phi_{1,0}}}{\sin E};\;\sigma_{P_{1}}=\frac{\sigma_{P_{1,0}}}{\sin E};\;\sigma_{{\dot{\rho }}_{1}}=\frac{\sigma_{{\dot{\rho }}_{1,0}}}{\sin E};$$

E is the satellite elevation; $\sigma_{\Phi_{1,0}}$, $\sigma_{P_{1,0}}$, and $\sigma_{{\dot{\rho }}_{1,0}}$ are the standard deviations of the carrier phase, pseudorange, and pseudorange rate observation errors, respectively, which can be set according to the corresponding observation accuracy of the GNSS receiver.

### 2.2. INS-Aided IAR

INS-assisted IAR uses high-precision prior position information provided by the INS to assist the GNSS in obtaining a more precise ambiguity float solution, reducing the search range and improving its accuracy and search efficiency. In particular, when the GNSS signal is partially interrupted, the tightly coupled filter still predicts the parameter error and continuously corrects the navigation result. When the satellite signal is received again, the position and variance matrices obtained by the INS are used to initialize the GNSS navigation module and serve as additional observations. After the integer ambiguity parameters are solved, the GNSS observations and the integer ambiguities are inputted into the tightly coupled filter to update the measurement. In this study, we use the position information predicted by INS as virtual observations. The observation equation for the INS-assisted acquisition of GNSS ambiguity float solution is as follows:

(13) $$\begin{array}{c} \left[\begin{array}{c} L_{P_{1}}\\ L_{\phi_{1}}\\ 0 \end{array}\right]=\left[\begin{array}{cc} B & \underset{n-1\times m}{0}\\ B & -\lambda_{1}\cdot \underset{n-1\times m}{I}\\ \underset{3\times 3}{I} & \underset{3\times m}{0} \end{array}\right]\left[\begin{array}{c} \delta p_{r_{2}}\\ \nabla \Delta N \end{array}\right]+\left[\begin{array}{c} \varepsilon_{p_{1}}\\ \varepsilon_{\Phi_{1}}\\ \varepsilon_{INS} \end{array}\right] \end{array}$$

where I is the identity matrix:

$$L_{P}{}_{1}=\left[\begin{array}{c} \nabla \Delta P_{1}^{S_{1},ref}-\nabla \Delta \rho_{INS,0}^{S_{1},ref}\\ \nabla \Delta P_{1}^{S_{2},ref}-\nabla \Delta \rho_{INS,0}^{S_{2},ref}\\ \begin{array}{c} \vdots \\ \nabla \Delta P_{1}^{S_{n-1},ref}-\nabla \Delta \rho_{INS,0}^{S_{n-1},ref} \end{array} \end{array}\right],\;L_{\phi_{1}}=\left[\begin{array}{c} \lambda_{1}\nabla \Delta \varphi {\;}_{1}^{S_{1},ref}-\nabla \Delta \rho_{INS,0}^{S_{1},ref}\\ \lambda_{1}\nabla \Delta \varphi_{1}^{S_{2},ref}-\nabla \Delta \rho_{INS,0}^{S_{2},ref}\\ \begin{array}{c} \vdots \\ \lambda_{1}\nabla \Delta \varphi_{1}^{S_{n-1},ref}-\nabla \Delta \rho_{INS,0}^{S_{n-1},ref} \end{array} \end{array}\right]$$

To improve the ambiguity initialization efficiency and solution accuracy, after obtaining the ambiguity float point solution, we implement different IAR strategies for different navigation stages. In the initialization phase, the float solution of the double-difference wide-lane ambiguity is directly rounded, and the two nearest integer values of each ambiguity are combined with other ambiguity values. Each combination is considered in the error equation for the least-squares solution, and the combination with the smallest error σ is used to calculate the ratio of the second smallest $\sigma_{1}$ to the smallest error σ.

(14) $$\begin{array}{c} ratio=\frac{\sigma_{1}}{\sigma } \end{array}$$

when σ and the ratio meet certain conditions, the ambiguity is considered to be successfully fixed [32]. In the continuous navigation phase, the Lambda algorithm [33] is used to obtain the integer ambiguity solution. After the satellite signal is interrupted or the innovation vector in the filter measurement update becomes very large, the integer ambiguity must be reinitialized. The GNSS/INS integration module transmits the position estimated by the INS to the GNSS navigation module for IAR.

### 2.3. RTSS Algorithm Based on Extended Kalman Filter

**Figure 2:**
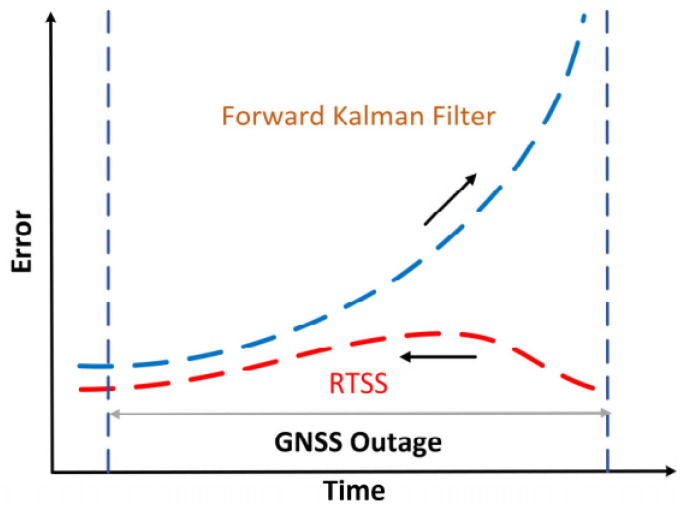
Rauch–Tung–Striebel smoothing (RTSS) algorithm diagram.

In the GNSS/INS integration system, the forward Kalman filter can only use historical information to estimate the current state, and it is susceptible to the influence of satellite signal interruption, which leads to great error accumulation. The RTSS algorithm can effectively limit the accumulation of pose errors by fully utilizing forward observations from the initial time to the current time and the backward dynamic constraint information from the end time to the current time [25]. Figure 2 displays the error curve processed by the RTSS when the GNSS signal is not locked. The error curve after the RTSS exhibits a rising and then a falling trend.

Reversing the RTSS algorithm based using forward Kalman filtering is conducted as follows. The forward Kalman filter solution is first used to obtain the state vector ${\hat{X}}_{f,k}$ of each sampling point, covariance matrix $P_{f,k}$, state transition matrix $\Phi_{k,k-1}$, prediction covariance matrix $P_{f,k/k-1}$, and navigation information obtained updating the INS strap-down algorithm. After forward filtering ends at the $t_{e}$ epoch, the RTSS algorithm is implemented in reverse order:

(15) $$\begin{array}{cc} K_{s,k} & =P_{f,k}\Phi_{k+1,k}^{T}P_{f,k+1/k}^{-1}\\ {\hat{X}}_{s,k} & ={\hat{X}}_{f,k}+K_{s,k}\left({\hat{X}}_{s,k+1}-{\hat{X}}_{f,k+1/k}\right)\\ P_{s,k} & =P_{f,k}-K_{s,k}\left(P_{f,k+1/k}-P_{s,k+1}\right)K_{s,k}^{T}\\ k & =t_{e}-1,t_{e}-2,\ldots ,2,1,0 \end{array}$$

where $P_{s,t_{e}}=P_{f,t_{e}}$, ${\hat{X}}_{s,t_{e}}={\hat{X}}_{f,t_{e}}$, and $K_{s,k}$ is the smooth gain matrix. Because the tightly coupled system uses a closed-loop feedback mechanism, when the pose information of the INS strap-down algorithm is updated and the random constant bias is corrected, the state vector ${\hat{X}}_{f,k}$ must be set to zero. Therefore, the state vector ${\hat{X}}_{f,k+1/k}$ of the one-step prediction is always  0. Similarly, after each RTSS, a closed-loop correction is also performed. This closed-loop correction minimizes the linearization error of the system model and ensures that the integration system is not influenced by divergence.

## 3. Results and Discussion

**Figure 3:**
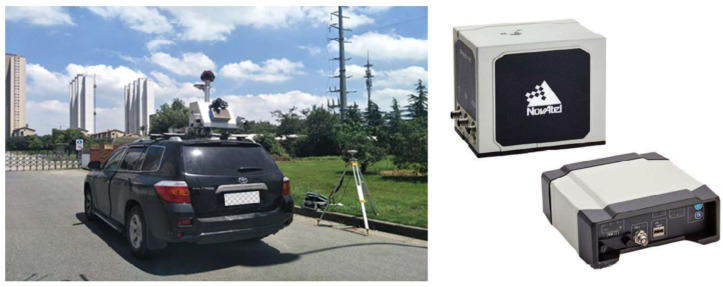
SPAN-LCI integrated navigation system.

To study the performance of the DGNSS/INS tightly coupled RTSS post-processing strategy in this study under the GNSS signal lock loss environment, we analyze and experimentally verified the following three aspects from the perspective of fully mining observations: (1) the ability of loosely coupled and tightly coupled to use observations in an environment with insufficient satellites, (2) the effectiveness of the RTSS algorithm during a period of GNSS signal interruption, and (3) the influence of INS-assisted IAR on the position and attitude accuracy in an environment with frequent lock loss. Experiments were conducted near Shandong University of Science and Technology (Qingdao, China). The split closed-loop fiber optic integrated navigation system SPAN-LCI manufactured by NovAtel (Figure 3) was used in the experiment, which contained a GNSS receiver and an IMU-LCI tactical fiber IMU. The primary performance indicators of SPAN-LCI [30] are displayed in Table 1. Before the experiment, the lever arm value was calibrated.

**Figure 4:**
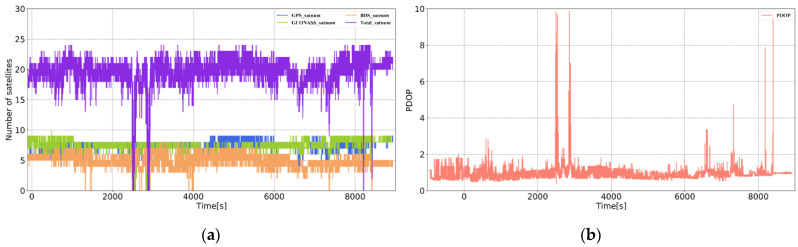

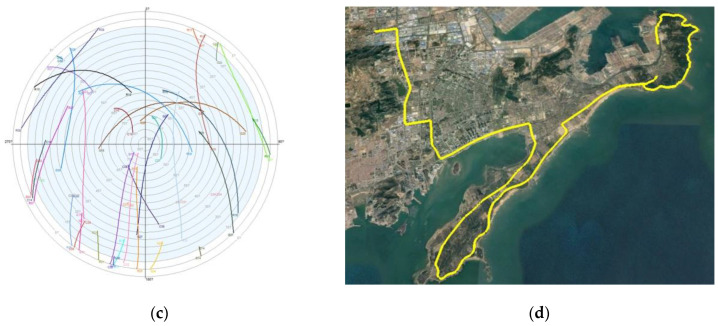
Experimental environment for simulating the satellite loss-of-lock experiment. (**a**) Number of common-view satellites of the base station and the rover station. (**b**) The position dilution of precision (PDOP) value of the rover station satellite. (**c**) Sky plot. (**d**) Experimental trajectory.

The GNSS receiver used was NovAtel ProPark6, which can realize the data collection of GPS, BDS, and GLONASS data. The sampling frequency was 20 Hz, and the GNSS antenna type was NOV703GGG. The reference station was set up at a known point in the marine survey comprehensive experimental field of Shandong University of Science and Technology, with an open surrounding environment. The data collection time of the mobile station was approximately 2.5 h, the baseline length typically exceeded 10 km, and the satellite suffered lock loss in several places. The experimental trajectory on Google Earth is displayed in Figure 4d. Combining the number of common-view satellites of the base station with the rover station in Figure 4a, the position dilution of precision (PDOP) of the rover station satellites (Figure 4b) and the sky plot (Figure 4c), further reflects the experimental environment. In the experiment, according to Table 1, the accelerometer random walk was set to $0.05{}^{\circ}/\sqrt{h}$, and the gyroscope random walk was set to $50\;\mu g/\sqrt{\mathrm{Hz}}$, which is used to set the system noise matrix. According to the observation accuracy of the GNSS receiver,$\;\sigma_{\Phi_{1,0}}$, $\sigma_{P_{1,0}}$ and $\sigma_{{\dot{\rho }}_{1,0}}$ were set to 0.005 m, 0.3 m, and 0.1 m, respectively. In this study, the NovAtel high-precision integrated navigation post-processing software Inertial Explorer 8.90 was used to perform DGNSS/INS tightly coupled smoothing in both directions, and the navigation result was used as a reference value.

### 3.1. Simulation Experiment

#### 3.1.1. Test 1

To analyze and verify the performance of the tightly coupled and loosely coupled systems in an environment where the number of available satellites is insufficient, experimental data within 6500 to 6560 epochs from the experimental data are intercepted. In a realistic urban environment, satellites with lower altitudes are more likely to be blocked, and satellites with higher altitudes are more suitable for simulating GNSS satellite loss-of-lock experiments. Therefore, G27, G26, and G16 were selected when simulating an observation environment with only three visible satellites, and G27 and G26 were selected when simulating an observation environment with only two visible satellites. What needs to be emphasized here is that the integer ambiguity of these selected satellites has been correctly fixed in the previous observation epoch.

**Figure 5:**
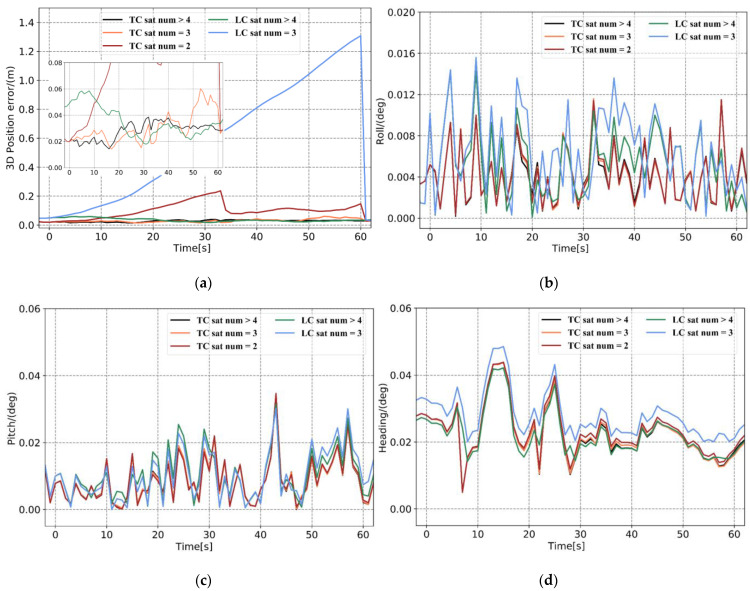
Root mean square error (RMSE) of tightly coupled and loosely coupled systems under different observable satellite numbers for 1 min: (**a**) 3D position error, (**b**) roll error, (**c**) pitch error, and (**d**) heading error.

In Figure 5 and Table 2, when there are sufficient satellites available, the accuracy of the position calculated by the loosely and tightly coupled systems are both at the centimeter level, and the attitude accuracy is at the same magnitude. Therefore, when the positioning accuracy of the GNSS is high, there is no significant difference between the solution results of the loosely and tightly coupled systems. When only three satellites are observed, the 3D position error calculated by loosely coupled increases exponentially over time and the maximum 3D position error reaches 1.3 m. Under the premise that the integer ambiguity is fixed correctly, the 3D position error calculated by tightly coupled system can be maintained at the centimeter level, and the root mean square error (RMSE) is 0.033 m. This result indicates that when the number of satellites is less than four, the tightly coupled system can still use the existing satellite observations for measurement updates to limit the error drift. However, the GNSS module in the loosely coupled system cannot use the existing three satellite observations to perform differential positioning solutions, which is equivalent to the GNSS signal being completely interrupted. When there are two observation satellites, the 3D position error calculated by tightly coupled reaches the decimeter level, because there is no redundant observation value.

In tightly coupled system, within a short period, the number of observable satellites substantially influences the position error, but the influence on the attitude error is not apparent. The RMSEs of the roll and pitch are 0.0058° and 0.0114°, respectively. The observability of the heading is poor, and when the number of observable satellites is sufficient, the RMSE is 0.0238°. The heading angle also conforms to the law that there are more available observations and sufficient accuracy. In general, when more observations are used, more accurate pose results can be obtained. Under the premise that the IAR is correct, the tightly coupled system can be measured and updated normally when there are fewer than four satellites, this can guarantee navigation solution accuracy. Therefore, the tightly coupled system is more suitable for observation environments where the satellite is partially locked.

#### 3.1.2. Test 2

To analyze the improvement effect of the RTSS algorithm on the navigation solution, this experiment performed loosely coupled forward filtering processing and loosely coupled reverse RTSS processing on simulated data in Test 1.

**Figure 6:**
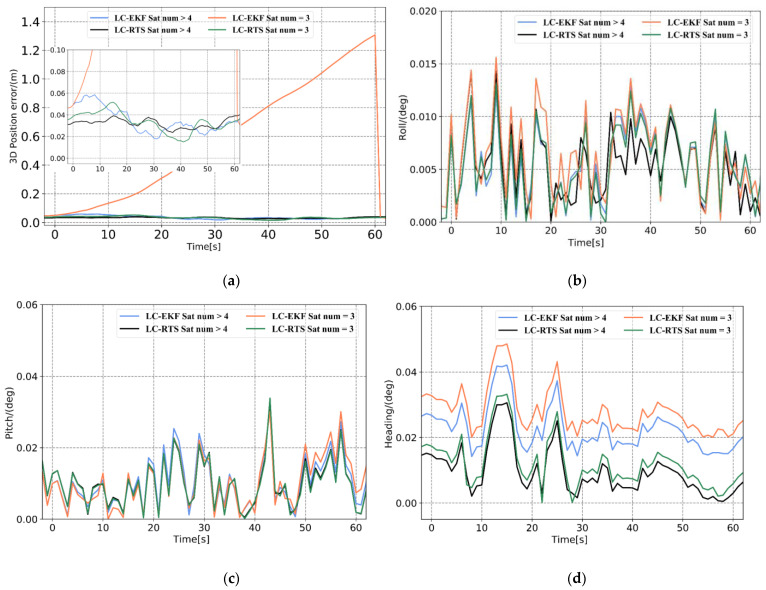
RMSEs under different processing methods. (**a**) 3D position error, (**b**) roll error, (**c**) pitch error, and (**d**) heading error.

The position and attitude error curves in Figure 6 and the error statistics in Table 3 demonstrate that the 3D position RMSE calculated by the loosely coupled system is 0.035 m after RTSS, which is 95% more accurate than the position solution before smoothing. Compared with real-time processing, RTSS is expected to bring accuracy improvement because more observations are used. The accuracy of the roll and pitch after RTSS improves slightly, by approximately 13% and 8%, respectively, and the error curve is smoother than that obtained by forward filtering alone. The most apparent improvement in attitude is that of the heading. It can be seen from Figure 6 that the heading after forward filtering exhibits a systematic error. After smoothing, the system error of the heading is significantly reduced by approximately 50%. Comparing the position and attitude error curves before and after smoothing, it can be seen that even if the GNSS signal is not interrupted, RTSS can improve the position and attitude accuracy and also effectively suppress the accumulation of INS estimation errors between measurement updates. Therefore, in the case of a partial or complete lock loss, RTSS as a bridging algorithm can sufficiently utilize the observations during the observation epoch before and after the lock loss, ensuring the accuracy of the navigation solution in the short term when the number of available satellites is insufficient.

#### 3.1.3. Test 3

In the partial satellite lock-out simulation experiments of Tests 1 and 2, satellites whose integer ambiguities have been correctly fixed in the previous epoch are selected. In an actual urban canyon environment, the satellite signal is received intermittently after the satellite signal is completely blocked for a short time, and the integer ambiguity must be fixed again. To analyze the performance of the INS-assisted IAR strategy in this study, we intercepted data within 374,470–374,560 epochs and designed four sets of experimental schemes.

Plan 1: The satellite signal is completely interrupted for 10 s and then continues to observe three satellites for 30 s.

Plan 2: The satellite signal is completely interrupted for 20 s and then continues to observe three satellites for 30 s.

Plan 3: The satellite signal is completely interrupted for 30 s and then continues to observe three satellites for 30 s.

**Figure 7:**
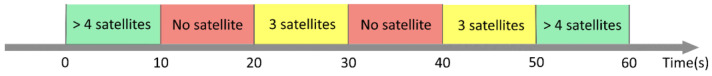
Plan 4 experimental situation.

Plan 4: First, the satellite signal is completely out of lock, and only three satellites are observed; finally, over four satellites are observed. The above three observation environments last for 10 s each. The specific experimental situation of plan4 is shown in Figure 7.

**Figure 8:**
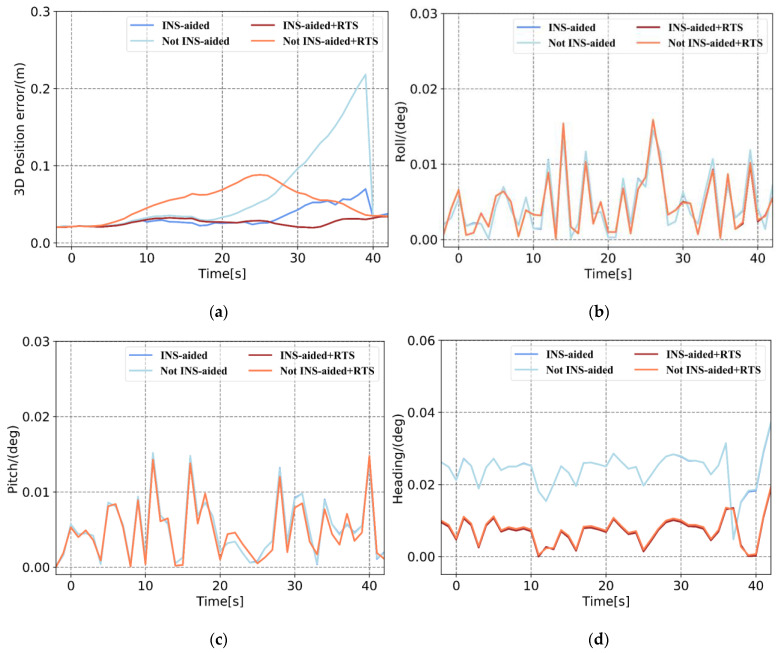
Plan 1 with and without inertial navigation system (INS)-aided pose error. (**a**) 3D position error, (**b**) roll error, (**c**) pitch error, and (**d**) heading error.

**Figure 9:**
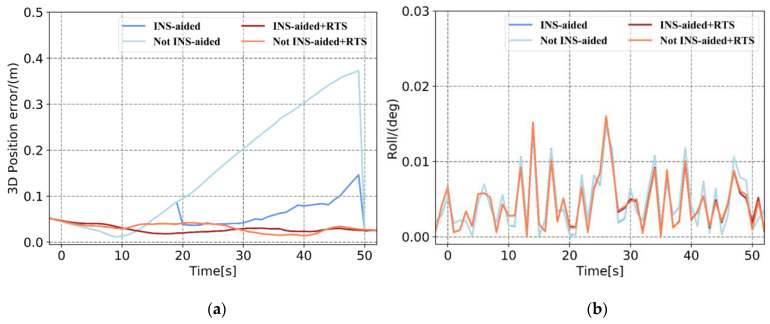

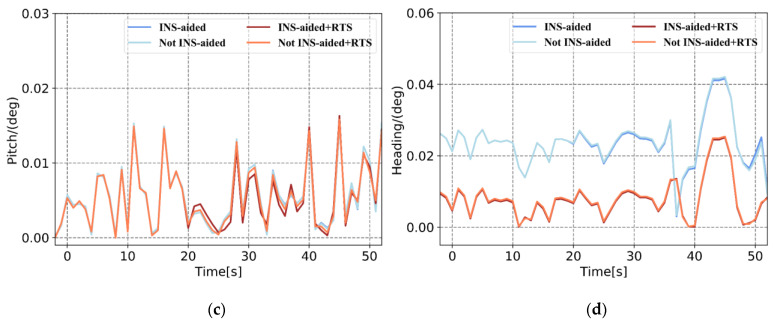
Plan 2 with and without INS-aided pose error. (**a**) 3D position error, (**b**) roll error, (**c**) pitch error, and (**d**) heading error.

**Figure 10:**
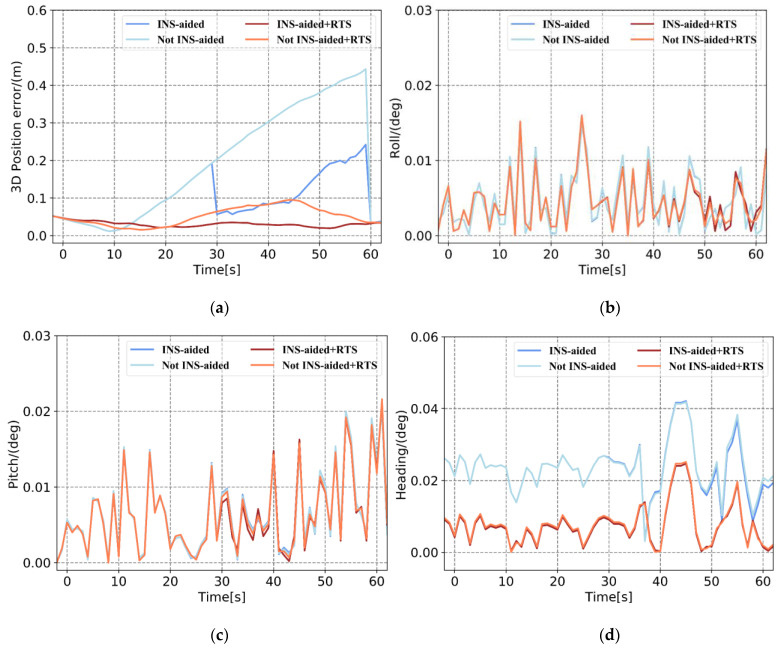
Plan 3 with and without INS-aided pose error. (**a**) 3D position error, (**b**) roll error, (**c**) pitch error, and (**d**) heading error.

Based on the pose errors in Figure 8, Figure 9 and Figure 10 and Table 4 and Table 5, it can be seen that when the position information calculated by the INS to assist the IAR is not used, the maximum 3D position error accumulates to 0.443 m, and the 3D position RMSE is 0.25 m in Plan 3. When using the prior position, the maximum position error is 0.242 m, and the RMSE is 0.117 m. When there is no INS-aided IAR, the ambiguity cannot be fixed. Therefore, even if three satellites are continuously observed after the satellite is interrupted, the observation values of these satellites cannot be used to update the measurement, resulting in the continuous accumulation of position errors. After using the prior position provided by the INS to assist the IAR, the integer ambiguities of the three satellites are quickly fixed. The observations of these three satellites are effectively used for measurement updates, and the increase in position error is significantly reduced. The accuracy of the attitude primarily depends on the inertial navigation, and the tactical-level inertial navigation is used in this experiment. Therefore, the influence of the INS-aided IAR on the attitude accuracy is not apparent. After RTSS, the 3D position RMSE within 1 min of the satellite signal interruption can be controlled within 0.06 m, and the attitude is also significantly improved. Specifically, the roll, pitch, and heading are improved by approximately 15%, 5%, and 60%, respectively.

In addition, after the satellite signal is interrupted for 10 s to 20 s, the position accuracy obtained after the INS-aided integer ambiguity is quickly fixed and remains at the centimeter level. After the satellite signal was interrupted for 30 s, the position error estimated by the INS reached the decimeter level. Even if the integer ambiguity was quickly fixed, and the measurement was updated, the position error still accumulated, and the final position error was at the decimeter level. The effectiveness of the INS-aided IAR therefore depends on the position accuracy of the INS estimation.

**Figure 11:**
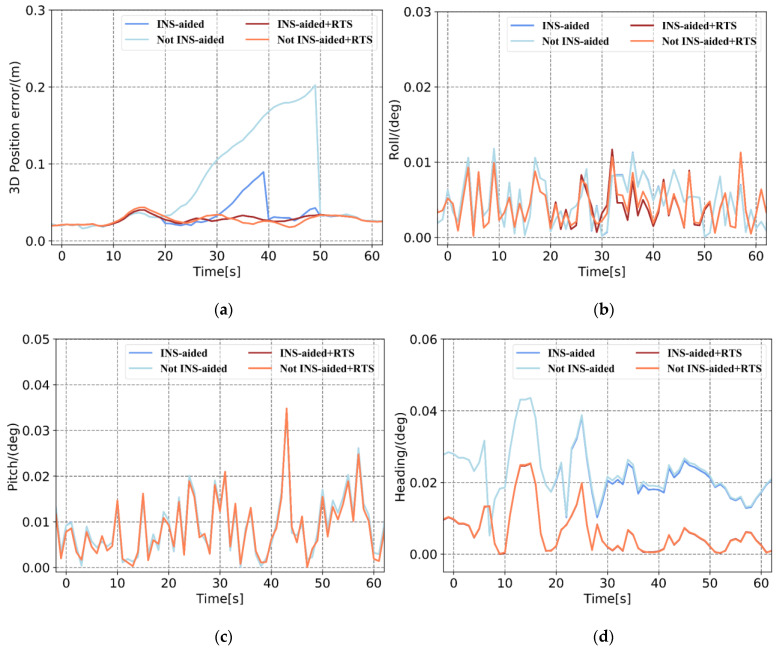
Comparison of Plan 4 with and without INS-aided pose error. (**a**) 3D position error, (**b**) roll error, (**c**) pitch error, and (**d**) heading error.

Based on Figure 11 and Table 4 and Table 5, it can be seen that in an environment with fewer than four satellites, intermittently observed after the satellite signal is interrupted, the position error calculated by the tightly coupled without INS-aided IAR accumulates. In contrast, for INS-assisted IAR, once there are observations, the position error is greatly reduced. Similarly, after smoothing, the accuracy of the position and posture also improved. Comparing the position error curves in Figure 8, Figure 9, Figure 10 and Figure 11 and the position error statistics in Table 4 and Table 5 indicates that the INS-aided IAR and smoothing post-processing tightly coupled strategy in this study does not require additional information, thereby fully utilizing the existing observations to maximize the limitation of the accumulation of errors and improve navigation solution accuracy.

### 3.2. Field Experiment in Urban Environment

**Figure 12:**
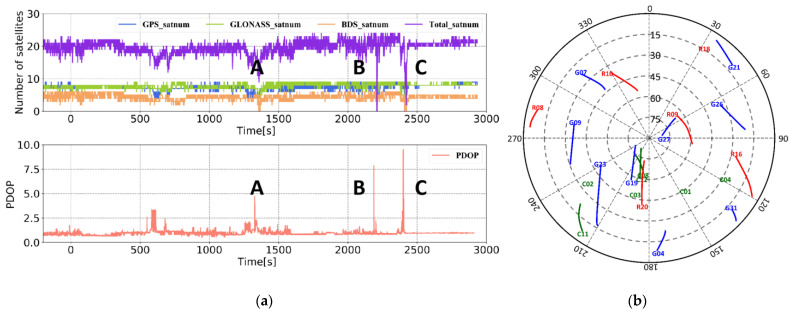
Experimental environment. (**a**) Number of common-view satellites of the base station and the rover station and PDOP of the rover station satellite. (**b**) Sky plot.

**Figure 13:**
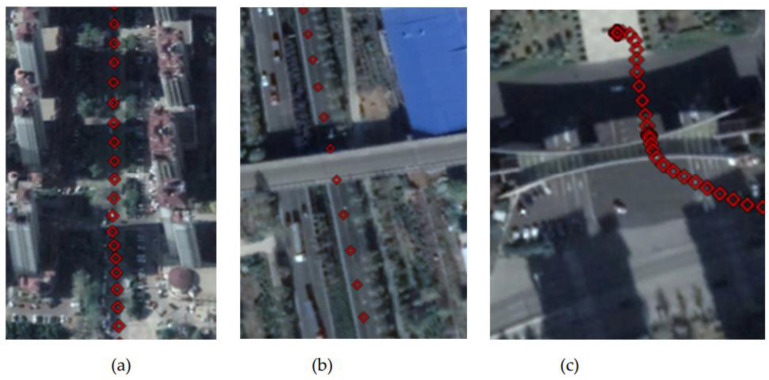
Experimental track of test vehicle. (**a**) Track at jumping point A. (**b**) Track at jumping point B. (**c**) Track at jumping point C.

**Figure 14:**
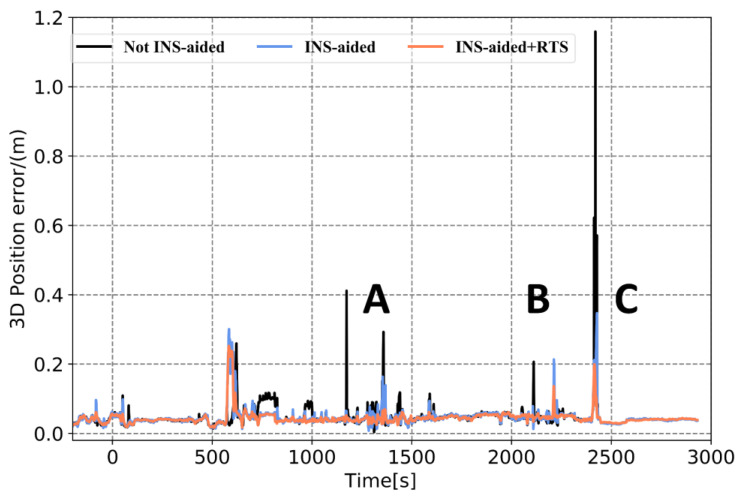
Comparison of 3D position RMSE obtained using three different methods.

**Figure 15:**
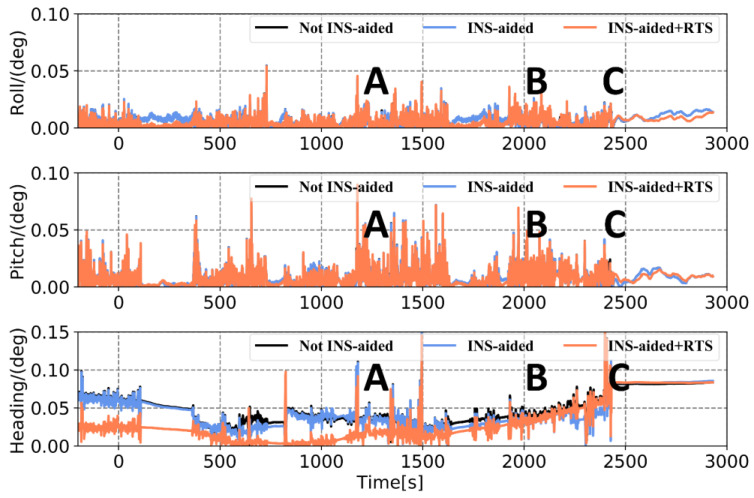
Comparison of attitude RMSE obtained using three different methods.

To verify the performance of the tightly coupled post-processing solution strategy in this study in an urban environment, data from the final hour of the Qingdao medium and a short baseline experiment were intercepted. Then, the tightly coupled solution without INS-aided IAR (Method 1), the tightly coupled solution with INS-aided IAR (Method 2), and the tightly coupled solution strategy in this study (Method 3) were used to process the data. Figure 12 displays the experimental observation environment. The total number of common-view satellites of the base and rover stations stabilized at approximately 16 in most periods, and the satellites frequently suffered lock loss during certain periods. The experimental trajectories at jumping points A, B, and C marked in Figure 12, Figure 13, Figure 14 and Figure 15 correspond to the three experimental trajectories in Figure 13. At jumping point A, there are tall buildings, and the satellite changes quickly. At this time, the observation structure is poor, and there are many integer ambiguity reinitializations. At jumping point B, the test vehicle passes a viaduct, causing the GNSS signal to suffer complete lock loss. When relocking the satellite signal, the satellite geometry is poor, and the observation accuracy is low. At jumping point C, the test vehicle passes through the south gate of Shandong University of Science and Technology, and the upper beam of the door is wide, and hence shields the GNSS signal.

In Figure 14 and Figure 15 and Table 6, it can be seen that when the observation environment and number of visible satellites are sufficient, the position errors acquired by the three solutions are maintained at the centimeter level. Of these, the position curve obtained using Method 3 is smoother, and the attitude accuracy is improved, the heading accuracy improvement is the greatest. At jumping points A, B, and C, the position and attitude accuracy are affected to varying degrees. The 3D position RMSE calculated using Method 1, 2, and 3, was 0.063 m, 0.054 m, and 0.049 m, respectively; meanwhile, the maximum error is 1.159 m, 0.374m, and 0.253m, respectively. Compared with the other two methods, the accuracy of the attitude calculated using Method 3, especially that of the heading, is significantly improved. Therefore, the solution strategy in this study effectively limits the increase in position and attitude errors when the satellite is out of lock by sufficiently utilizing the observations before and after the satellite signal occlusion period. This is an effective means to improve the positioning and attitude accuracy of the GNSS/INS integrated system in an urban environment.

## 4. Conclusions

In an actual urban environment, due to the influence of tall buildings, trees, and other occlusion factors, the satellite signal frequently suffers lock loss, causing a reduction in the accuracy of the GNSS/INS integrated system, rendering it unable to meet application requirements. This study implemented an INS-aided IAR DGNSS-INS tightly coupled smoothing post-processing strategy. This strategy uses INS-aided IAR in the ambiguity initialization stage after the GNSS signal is interrupted to achieve fast IAR, followed by RTSS to determine the navigation solution during lock-out period processing. Moreover, this solution strategy does not require additional observations but improves the accuracy of the integrated system by fully utilizing existing observations. The primary results obtained through experimental analysis in this study are as follows.

(1) The performances of the loosely and tightly coupled systems and the improvement effect of the RTSS algorithm on the navigation solution were analyzed through simulation experiments where available satellites is insufficient. The experimental results demonstrate that when the number of observable satellites was sufficient, there is no significant difference in the positioning and attitude determination performance of the loosely and tightly coupled satellites. When the number of observation satellites was less than four, compared to the loosely coupled system, the tightly coupled system fully utilized the observations of satellites to update the measurements and constrain the drift error of the inertial device. In addition, during the lock-out period, the accuracy of the loosely coupled system after RTSS was significantly improved. The position accuracy was improved by approximately 95%. The degree of improvement in the roll and pitch was approximately 10%, and that in the heading was approximately 50%.

(2) The performances of the tightly coupled system without INS-aided IAR, the tightly coupled system with INS-aided IAR, and the solution strategy were compared and analyzed through simulation and field experiments. The results demonstrate that the strategy proposed in this study can use the position provided by the INS to quickly fix integer ambiguity of the three satellites after the satellite signal is completely interrupted, allowing the observations of the three satellites to become available observations. The strategy in this study effectively limited the growth of the pose error, and the position and attitude accuracy of the navigation solution was further improved after smoothing.

In this study, the performance of the tightly coupled DGNSS/INS under the condition of short-term satellite signal multi-occlusion was analyzed, and solutions were provided. However, it was difficult to obtain centimeter-level positioning accuracy with GNSS signals that are completely out of lock for a long period. Future work should focus on special environments where GNSS is completely out of lock for a long period, such as garages and tunnels, to further enrich quality control methods and to obtain more reliable and accurate navigation information.

## Figures and Tables

**Table 1:** SPAN-LCI performance indicators.

	Gyroscope	Accelerometer
Bias stability	<1.0°/h	<1.0 mg
Random walk	$<0.05{}^{\circ}/\sqrt{h}$	$50\;\mu g/\sqrt{\mathrm{Hz}}$
Scale factor	100 ppm	250 ppm
Sampling rate	200 Hz	200 Hz

**Table 2:** RMSE statistics of 3D position and attitude of the tightly coupled and loosely coupled systems under 1 min of different observable satellite numbers.

		Tightly Coupled (TC)			Loosely Coupled (LC)	
Number of Satellites		>4	3	2	>4	3
Position Error (m)	3D	0.028	0.033	0.115	0.036	0.714
Attitude Error (°)	Roll	0.0058	0.0058	0.0058	0.0066	0.0076
	Pitch	0.0114	0.0114	0.0114	0.0126	0.0128
	Heading	0.0238	0.0239	0.0250	0.0235	0.0289

**Table 3:** Position and attitude RMSEs under different processing methods.

		Satellite Number ≥ 4		Satellite Number = 3	
		LC-EKF	LC-RTS	LC-EKF	LC-RTS
Position Error (m)	3d	0.036	0.032	0.714	0.035
Attitude Error (°)	Roll	0.0066	0.0063	0.0076	0.0066
	Pitch	0.0126	0.0119	0.0128	0.0118
	Heading	0.0235	0.0121	0.0289	0.0143

**Table 4:** Maximum error (MAX) and RMSE of position statistics for different plans.

	RMSE (m)				MAX (m)			
	INS-Aided		Not INS-Aided		INS-Aided		Not INS-Aided	
	EKF	RTS	EKF	RTS	EKF	RTS	EKF	RTS
Plan 1	0.036	0.027	0.085	0.057	0.070	0.032	0.218	0.088
Plan 2	0.062	0.029	0.203	0.032	0.146	0.044	0.373	0.043
Plan 3	0.117	0.030	0.250	0.057	0.242	0.044	0.443	0.095
Plan 4	0.037	0.028	0.098	0.029	0.090	0.040	0.202	0.044

**Table 5:** RMSE of attitude for different plans.

		INS-Aided		Not INS-Aided	
		EKF	RTS	EKF	RTS
Roll (°)	Plan 1	0.0059	0.0056	0.0059	0.0056
	Plan 2	0.0062	0.0058	0.0062	0.0059
	Plan 3	0.0061	0.0057	0.0061	0.0057
	Plan 4	0.0058	0.0049	0.0058	0.0050
Pitch (°)	Plan 1	0.0070	0.0067	0.0070	0.0068
	Plan 2	0.0082	0.0079	0.0082	0.0079
	Plan 3	0.0087	0.0082	0.0087	0.0082
	Plan 4	0.0114	0.0110	0.0114	0.0110
Heading (°)	Plan 1	0.0247	0.0099	0.0246	0.0096
	Plan 2	0.0247	0.0100	0.0249	0.0099
	Plan 3	0.0267	0.0111	0.0271	0.0114
	Plan 4	0.0238	0.0089	0.0242	0.0090

**Table 6:** Position and attitude error statistics obtained using three different methods.

	RMSE(m)			MAX(m)		
	Not INS-Aided	INS-Aided	INS-Aided RTS	Not INS-Aided	INS-Aided	INS-Aided RTS
Position	0.063	0.054	0.049	1.159	0.347	0.253
Roll	0.0085	0.0085	0.0072	0.0546	0.0543	0.0539
Pitch	0.0115	0.0115	0.0113	0.0883	0.0886	0.0897
Heading	0.0510	0.0490	0.0425	0.1684	0.1541	0.1679

## Data Availability

The data presented in this study are available on request from the corresponding author. The data are not publicly available due to project restrictions.

## Appendix A

### Appendix A.1. Attitude Error Equation

The real direction cosine matrix (DCM) of ECEF frame relative to IMU body frame is $C_{b}^{e}$ and the estimated DCM is ${\tilde{C}}_{b}^{e}$. There is a matrix $C_{e}^{e^{\prime }}$ that can realize the transformation from real DCM to estimated DCM, which is given as [34]

(A1) $$\begin{array}{c} {\tilde{C}}_{b}^{e}=C_{e}^{e^{\prime }}C_{b}^{e} \end{array}$$

where the small angle approximation applies, $C_{e}^{e^{\prime }}$ can be approximately expressed as

(A2) $$\begin{array}{c} C_{e}^{e^{\prime }}\approx I-\left[\phi \times \right] \end{array}$$

where I is the identity matrix; ϕ is the attitude error vector; (a)× represents a skew-symmetrical matrix composed of vector a. Substituting Equation (A2) into Equation (A1),

(A3) $$\begin{array}{c} {\tilde{C}}_{b}^{e}=\left(I-\left[\phi \times \right]\right)C_{b}^{e} \end{array}$$

Differentiate Equation (A3),

(A4) $$\begin{array}{c} {\dot{\tilde{C}}}_{b}^{e}=\left[-\dot{\phi }\times \right]C_{b}^{e}+\left(I-\left[\phi \times \right]\right){\dot{C}}_{b}^{e} \end{array}$$

where · is the differential symbol. The real attitude differential equation is given as

(A5) $$\begin{array}{c} {\dot{C}}_{b}^{e}=C_{b}^{e}\left[\omega_{ib}^{b}\times \right]-\left[\omega_{ie}^{e}\times \right]C_{b}^{e} \end{array}$$

The attitude differential equation including error is given as

(A6) $$\begin{array}{c} {\dot{\tilde{C}}}_{b}^{e}={\tilde{C}}_{b}^{e}\left[{\tilde{\omega }}_{ib}^{b}\times \right]-\left[{\tilde{\omega }}_{ie}^{e}\times \right]{\tilde{C}}_{b}^{e} \end{array}$$

where $\omega_{ie}^{e}$ is the earth rotation; ${\tilde{\omega }}_{ie}^{e}$ is measurement value of the earth rotation; $\omega_{ib}^{b}$ is angular rate; ${\tilde{\omega }}_{ib}^{b}$ is measurement value of angular rate,

(A7) $$\begin{array}{c} {\tilde{\omega }}_{ib}^{b}=\omega_{ib}^{b}+\delta \omega_{ib}^{b} \end{array}$$

where $\delta \omega_{ib}^{b}$ is the measurement error of gyroscope. The results of Equation (A4) and Equation (A6) should be completely equivalent,

(A8) $$\begin{array}{c} \left[-\dot{\phi }\times \right]C_{b}^{e}+\left(I-\left[\phi \times \right]\right){\dot{C}}_{b}^{e}={\tilde{C}}_{b}^{e}\left[{\tilde{\omega }}_{ib}^{b}\times \right]-\left[{\tilde{\omega }}_{ie}^{e}\times \right]{\tilde{C}}_{b}^{e} \end{array}$$

Substituting Equations (A3), (A5) and (A7) into Equation (A8), and the error of ${\tilde{\omega }}_{ie}^{e}$ and the second order small quantity of error are ignored in practical operation [28]. The attitude error equation is obtained:

(A9) $$\begin{array}{c} \dot{\phi }=-\left[\omega_{ie}^{e}\times \right]\phi -C_{b}^{e}\delta \omega_{ib}^{b} \end{array}$$

### Appendix A.2. Velocity Error Equation

The velocity error is expressed as

(A10) $$\begin{array}{c} \delta v^{e}={\tilde{v}}^{e}-v^{e} \end{array}$$

where $\delta v^{e}$ is velocity error vector; ${\tilde{v}}^{e}$ is the velocity estimated by INS; $v^{e}$ is the real velocity. Differentiate Equation (A10),

(A11) $$\begin{array}{c} \delta {\dot{v}}^{e}={\dot{\tilde{v}}}^{e}-{\dot{v}}^{e} \end{array}$$

The real velocity differential equation is given as

(A12) $$\begin{array}{c} {\dot{v}}^{e}=C_{b}^{e}f_{ib}^{b}-2\left[\omega_{ie}^{e}\times \right]v^{e}+g^{e} \end{array}$$

The velocity differential equation including error is given as

(A13) $$\begin{array}{c} {\dot{\tilde{v}}}^{e}={\tilde{C}}_{b}^{e}{\tilde{f}}_{ib}^{b}-2\left[{\tilde{\omega }}_{ie}^{e}\times \right]{\tilde{v}}^{e}+{\tilde{g}}^{e} \end{array}$$

where $g^{e}$ is the gravity vector; ${\tilde{g}}^{e}$ is measurement value of gravity; $f_{ib}^{b}$ is the specific force; and ${\tilde{f}}_{ib}^{b}$ is the measurement value of the specific force,

(A14) $$\begin{array}{c} {\tilde{f}}_{ib}^{b}=f_{ib}^{b}+\delta f_{ib}^{b}\\ {\tilde{g}}^{e}=g^{e}+\delta g^{e} \end{array}$$

where $\delta f_{ib}^{b}$ is the measurement error of accelerometers; $\delta g^{e}$ is the gravity error vector. Substituting Equation (A12), (A13) and (A14) into Equation (A11), the error of ${\tilde{\omega }}_{ie}^{e}$ and the second order small quantity of error are ignored in practical operation. The velocity error equation is obtained:

(A15) $$\begin{array}{c} \delta {\dot{v}}^{e}=C_{b}^{e}\delta f_{ib}^{b}+\left[\left(C_{b}^{e}f_{ib}^{b}\right)\times \right]\phi -2\left[\omega_{ie}^{e}\times \right]\delta v^{e}+\delta g^{e} \end{array}$$

### Appendix A.3. Position Error Equation

The position error is expressed as

(A16) $$\begin{array}{c} \delta r^{e}={\tilde{r}}^{e}-r^{e} \end{array}$$

where $\delta r^{e}$ is position error vector; ${\tilde{r}}^{e}$ is the position estimated by INS; $r^{e}$ is the real position. Differentiate Equation (A16),

(A17) $$\begin{array}{c} \delta {\dot{r}}^{e}=\dot{\tilde{r}}-{\dot{r}}^{e} \end{array}$$

The real position differential equation is given as

(A18) $$\begin{array}{c} {\dot{r}}^{e}=v^{e} \end{array}$$

The position differential equation including error is given as

(A19) $$\begin{array}{c} {\dot{\tilde{r}}}^{e}={\dot{\tilde{v}}}^{e} \end{array}$$

Substituting Equations (A18) and (A19) into Equation (A17), and the position error equation is obtained:

(A20) $$\begin{array}{c} \delta {\dot{r}}^{e}=\delta v^{e} \end{array}$$

In conclusion, the INS error equation in ECEF are as follows:

(A21) $$\begin{array}{c} \left(\begin{array}{c} \dot{\phi^{e}}\\ \begin{array}{c} \delta {\dot{v}}^{e}\\ \delta {\dot{r}}^{e} \end{array} \end{array}\right)=\left(\begin{array}{c} -\left[\omega_{ie}^{e}\times \right]\phi^{e}-C_{b}^{e}\delta \omega_{ib}^{b}\\ \begin{array}{c} C_{b}^{e}\delta f_{ib}^{b}+\left[\left(C_{b}^{e}f_{ib}^{b}\right)\times \right]\phi^{e}-2\left[\omega_{ie}^{e}\times \right]\delta v^{e}\\ \delta v^{e} \end{array} \end{array}\right) \end{array}$$
